# Monoclonal Immunotactoid Glomerulopathy Associated With Chronic Lymphocytic Leukemia: A Case Report

**DOI:** 10.7759/cureus.96452

**Published:** 2025-11-09

**Authors:** Gholamreza Badiee, Atefeh Kalantary, Sina Shafiei, Jean Hou, Michael E Lazarus

**Affiliations:** 1 Internal Medicine, David Geffen School of Medicine at UCLA (University of California, Los Angeles), Los Angeles, USA; 2 Pathology and Laboratory Medicine, Cedars-Sinai Medical Center, Los Angeles, USA

**Keywords:** chronic lymphocytic leukemia (cll), immunotactoid glomerulopathy, monoclonal gammopathy of renal significance, monoclonal gammopathy of undetermined significance (mgus), nephrotic range proteinuria

## Abstract

Immunotactoid glomerulopathy (ITG) is exceedingly rare in clinical practice. A majority of cases are associated with an underlying hematological disorder and specifically chronic lymphocytic leukemia (CLL). The treatment consists of managing the underlying disease and supportive therapy. We present the case of an 80-year-old patient with a history of CLL who presented with proteinuria and acute kidney injury and eventually developed hematuria. His renal biopsy revealed monoclonal ITG. He began a course of chemotherapy for CLL and achieved remission with improved renal function.

## Introduction

Immunotactoid glomerulopathy (ITG) is a rare form of glomerulonephritis that is found in less than 0.1% of all renal biopsies on native kidneys. It is often associated with an underlying overt or undiagnosed hematological disorder specifically affecting plasma cells or lymphocytes [1]. Common clinical presentations include the combination of hypertension, significant proteinuria, hematuria, and acute kidney injury (AKI). Underlying blood dyscrasias are present in over 65% of ITG patients. Of this, 41% are chronic lymphocytic leukemias (CLL) and small lymphocytic lymphoma (SLL). Less than one-fifth of cases have coexistent monoclonal gammopathy or multiple myeloma [1-3].

## Case presentation

An 80-year-old man with a past medical history of CLL, hypertension (HTN), atrial flutter, and normocytic anemia was admitted with progressive shortness of breath. Symptoms developed over the prior six weeks. He noticed worsening dyspnea after he was diagnosed with atrial flutter three months prior. He saw his cardiologist, who referred him for a curative ablation procedure. Unfortunately, his shortness of breath progressed to the point that he was unable to perform activities of daily living without significant fatigue and frequent rest. He reported three-pillow orthopnea, increasing swelling of his lower extremities, and abdominal distention over the prior two weeks. He denied chest pain, fever, chills, nausea, vomiting, or diarrhea. 

Upon arrival in the emergency department, his blood pressure (BP) was 170/90 mmHg, heart rate (HR) 57 beats per minute, and respiratory rate (RR) 22 breaths per minute. He was afebrile with an oxygen (O2) saturation of 92% on room air. His physical exam was notable for jugular venous pressure of 11 cm with a normal carotid upstroke. His lung exam had normal chest excursion bilaterally, stony dullness to percussion at both his lung bases, and decreased bilateral breath sounds over the lower lung with scattered wet crackles on auscultation. Lower extremity edema of 2+ was noted bilaterally with no appreciable calf tenderness. His chest x-ray (CXR) showed small-to-moderate bibasilar opacities compatible with pleural effusions and adjacent atelectasis. Electrocardiogram (EKG) revealed sinus bradycardia (HR 55 bpm) with no acute ST or T-wave changes. He was given aspirin 325 mg x 1 and 0.4mg of sublingual nitroglycerin for possible acute coronary syndrome rule out. Subsequent troponin levels normalized.

The patient was admitted to the hospital for volume overload and AKI. He was started on Lasix 20 mg intravenously (IV) twice daily and increased to 40 mg IV twice daily. His echocardiogram showed a left ventricular ejection fraction (LVEF) of 55-60% and a mildly reduced right ventricular ejection fraction. Renal ultrasound was unremarkable without evidence of obstructive uropathy. A venous Doppler scan of the lower extremities was negative for deep vein thrombosis (DVT). His Lexi-scan stress test was negative for acute cardiac ischemia but notable for a small, fixed perfusion defect in the inferior left ventricular wall, suggestive of prior infarction with no reversibility. His 24-hour urine protein was noted to be significantly elevated, and his serum albumin was below the normal range. His laboratory and serologic results are shown in Table 1. An acute hepatitis panel was negative, and complement studies showed a low C3 and a C4 within normal range. Both perinuclear and cytoplasmic anti-cytoplasmic antibodies (pANCA and cANCA) were negative. His serum protein electrophoresis (SPEP) revealed an M-spike in the gamma region. Subsequent serum protein immunofixation electrophoresis (IFE) revealed a normal serum free kappa light chain and an elevated serum free lambda light chain.

**Table 1:** Laboratory test results at baseline, presentation, and two months post treatment c-ANCA: antineutrophil cytoplasmic autoantibody, cytoplasmic; p-ANCA: perinuclear anti-neutrophil cytoplasmic antibodies; SPEP: serum protein electrophoresis; ANA: antinuclear antibody

Laboratory Test	Result at Presentation	Baseline (6 months prior)	8-weeks post treatment	Normal Range
White Cell Count (WCC)	44.9 × 10^9^/L	21 × 10^9^/L	-	4.5 to 11.0 × 10^9^/L
Hemoglobin	10.9 g/dl	13 g/dl	-	14 to 18 g/dl
Platelets	105 per μL	200 per μL	-	150,000-450,000 per μL
Blood Urea Nitrogen (BUN)	32 mg/dL	14 mg/dL	17 mg/dL	6-20 mg/dL
Creatinine	1.89 mg/dL	0.9 mg/dL	1.1mg/dL	0.6-1.2 mg/dL
Serum Albumin	3.1 g/dL	-	-	3.5 to 5.0 g/dL
Beta-Natriuretic Peptide (BNP)	351 pg/mL	-	-	<100 pg/mL
High Sensitivity Troponin I	22 ng/L	-	-	<20 ng/L
MCV (Mean Cell Volume)	96 fL	90 fL	-	80-100 fL
Hepatitis Panel	Negative	-	-	Negative
C3 (Complement component 3)	59 mg/dL	-	-	88-200 mg/dL
C4 (Complement component 4)	12 mg/dL	-	-	88 to 201 (mg/dL)
c-ANCA	Negative	-	-	Negative
p-ANCA	Negative	-	-	Negative
SPEP	M-spike in gamma region	-	-	Normal
Free kappa light Chains (IFE)	11 mg/L	-	-	3.3 to 19.4 mg/L
Free lambda light Chains (IFE)	32 mg/L	-	-	5.7 to 26.3 mg/L.
Cryoglobulins	Negative	-	-	Negative
Spot Urine protein	-	-	180mg	<150mg per day
Urinalysis	+++ Protein/Blood	-	-	Negative
Urine 24-hour protein collection	5.6 g/day	-	-	< 150 mg perday
ANA	<1:40	-	-	<1:40 dilution

**Figure 1:**
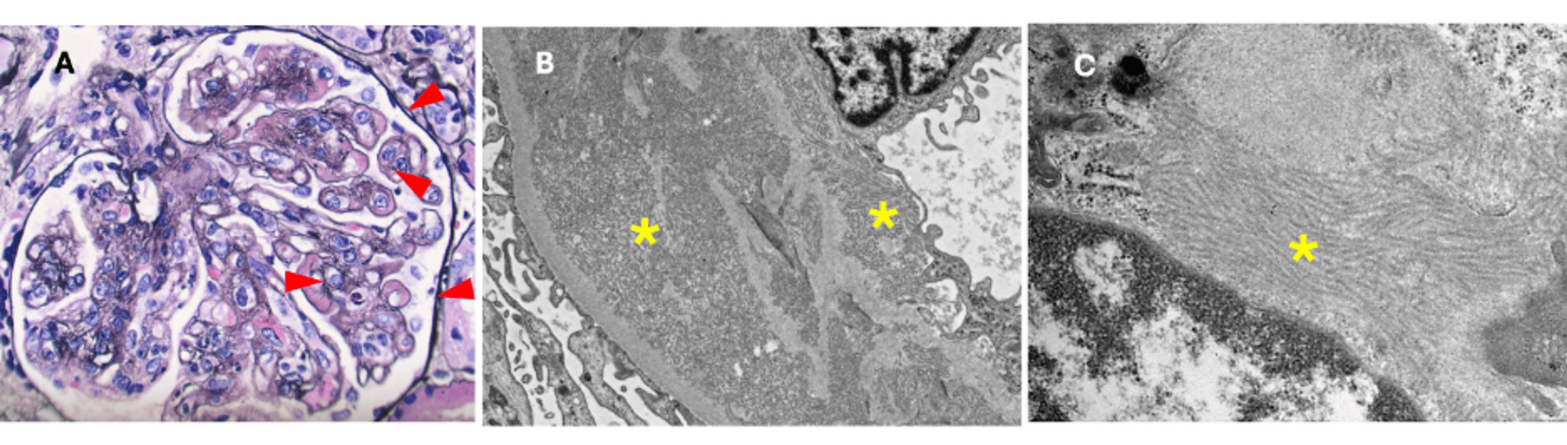
A. Light microscopy reveals glomeruli with mesangial and endocapillary hypercellularity, and numerous subendothelial immune complex deposits (arrowhead), Jones methenamine silver stain, magnification 400X. B. Electron microscopy reveals frequent subendothelial deposits with an organized microtubular substructure (asterisk), magnification 6,000X. C. The microtubular deposits are primarily subendothelial and mesangial, and are organized into “streaming”, parallel bundles (asterisk), magnification 20,000X.

During his hospital stay, the patient developed gross hematuria, and on evaluation by Urology, was found to have no bladder or urethral lesions or stones. His hemoglobin and platelets trended downward initially, but he did not require blood product transfusion. He underwent computed tomography (CT) guided biopsy of the left kidney. The pathology results confirmed immunoglobulin G-1 (IgG) lambda-restricted immune complex glomerulonephritis with organized microtubular substructures, most consistent with immunotactoid glomerulonephritis, acute tubular necrosis, and severe arteriosclerosis (Figure 1). The histopathological glomerular lesion is a membranoproliferative glomerulopathy.

**Figure 2:**
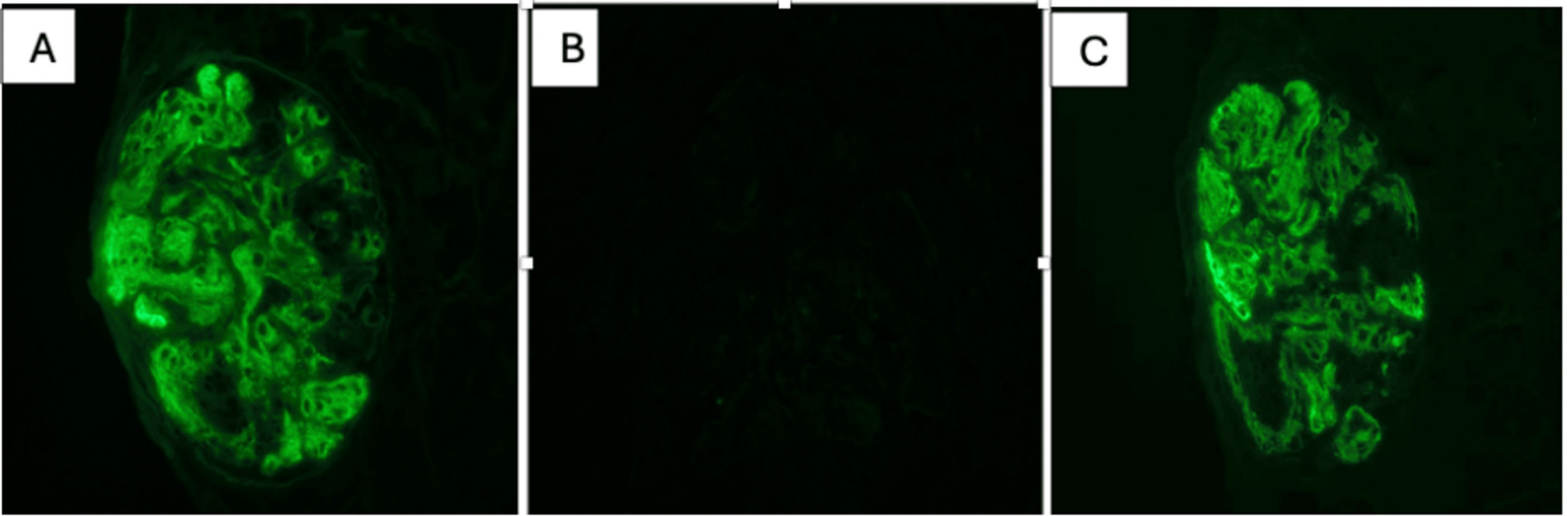
A. Immunofluorescence microscopy revealed mesangial and capillary wall IgG staining in a global distribution (magnification 400X). B. Staining control for kappa light chain was negative (magnification 400X). C. The deposits displayed lambda light chain restriction in a pattern and distribution correlating with the IgG staining (magnification 400X).

Immunofluorescence microscopy revealed mesangial and capillary wall IgG staining in a global distribution (Figure 2A). The biopsy results confirmed monoclonal gammopathy of renal significance (MGRS), most likely associated with his known diagnosis of CLL. He was started on the Bruton's tyrosine kinase (BTK) inhibitor zanubrutinib 160 mg twice a day to treat his leukemia and achieved remission of his CLL and improved renal function.

The patient will continue to take oral zanubrutinib, 160 mg twice daily, continuously until evidence of disease progression or drug-associated toxicities develops. This dosing is supported by National Comprehensive Cancer Network guidelines (NCCN). Dose modifications may be required for adverse reactions such as hepatotoxicity or drug interactions. The follow-up period and disease monitoring include regular clinical evaluation and laboratory checks. We drew labs every two weeks initially. Based on our patient’s clinical response after two months of therapy, his monitoring switched to monthly labs or as clinically indicated. The main laboratory tests we monitored are CBC with differential to detect neutropenia, thrombocytopenia, anemia, and lymphocyte counts, as cytopenias are common adverse effects of the drug. Ongoing renal function is assessed with serum creatinine, blood urea nitrogen (BUN), which is especially important in patients with renal dysfunction. Hepatotoxicity is a common side effect necessitating regular transaminase, alkaline phosphatase, and serum bilirubin checks. We rechecked a urinalysis and proteinuria quantification to determine the kidneys' response to treatment at week five. Immunoglobulin levels and serum protein electrophoresis will be rechecked to assess for monoclonal protein response to therapy. A multidisciplinary team consisting of an oncologist, nephrologist, and primary care physician is involved in coordinating, planning, and monitoring care. His renal function and blood counts have been stable, and his primary doctor will likely follow his labs and clinical care as he recovers.

## Discussion

ITG is subdivided into two subtypes, monoclonal and polyclonal variants. In 35% of the polyclonal variants, the anti-neutrophil antibody (ANA) is positive. In one-fourth of cases, it is associated with an underlying autoimmune disease [2]. Monoclonal ITG, as seen in this case, has a higher incidence of associated lymphoma, 53% as opposed to 11% in the polyclonal form. A smaller percentage of monoclonal ITG is associated with multiple myeloma (8%) and 22% with monoclonal gammopathy [3,4].

ITG in the setting of CLL is a well-defined association. A retrospective study by Nasr et al., reviewing 73 immunotactoid patients, found that the incidence of an abnormal serum free light chain ratio was higher than might be expected in the general population, as seen in our patient [2]. This was associated with 40% of patients progressing to end-stage renal disease. Unfortunately, recurrence of ITG following kidney transplantation is common [5]. It is also worth noting that the polyclonal ITG variants are treated less aggressively and tend to present with a high serum creatinine level. Polyclonal ITG demonstrates biopsy findings of a more advanced tubulointerstitial scarring compared to patients with monoclonal ITG [6].

Definitive diagnosis of ITG is made by renal biopsy and electron microscopy. Deposits of organized microtubular immunoglobulin are the hallmark finding and are usually seen in parallel arrays (Figure 1C). The more common monoclonal variety has predominantly IgG light chain deposits seen on immunofluorescence, as in this case (Figure 2A). 

The term monoclonal gammopathy of renal significance (MGRS) was coined in 2012 by the International Kidney and Monoclonal Gammopathy Research Group (IKMG). Their goal was to differentiate it from monoclonal gammopathy of undetermined significance (MGUS) [7]. Both conditions have circulating monoclonal immunoglobulins arising from innate B cells or plasma cells, but insufficient additional criteria to qualify for a diagnosis of multiple myeloma [7]. Over the last two decades, it became apparent that monoclonal immunoglobulins are inherently nephrotoxic and not necessarily dependent on the severity of the associated hematologic condition responsible for the abnormal clones. Hence, there is a need for a new, separate diagnostic MGRS classification providing guidelines for the treatment of the underlying hematologic disorder. Renal biopsy studies have shown that almost 50% of MGUS patients who get a kidney biopsy have MGRS-related conditions. These include abnormal serum free light chain ratios, proteinuria, and microscopic hematuria as common hallmarks [8]. Other than ITG and CLL, other hematologic disorders and their associated renal conditions are under the MGRS classification. These include (but are not limited to) immunoglobulin-related amyloidosis with nephrotic syndrome, cryoglobulinemic glomerulonephritis type 1 with lymphoplasmacytic lymphoma, and light chain proximal tubulopathy and multiple myeloma [9].

Prior to the current admission, the patient's CLL did not meet criteria for active treatment. These are progressive anemia and/or thrombocytopenia, rising lymphocytosis (>5000 leukemic cells per cubic millimeter), and clinical symptoms from splenomegaly/lymphadenopathy or both. After diagnosis of his ITG and MGRS, he was a candidate for treatment of the underlying CLL with tyrosine kinase inhibitors. Additional therapy can include immune suppression, managing associated conditions such as his hypertension and proteinuria with angiotensin converting enzyme inhibitors or angiotensin receptor blockers. Volume overload in the acute phase responded to diuresis with IV furosemide. Our patient's condition improved after treatment directed at the malignant CLL clones. Targeted therapy has been shown to have higher rates of renal response and remission, especially in monoclonal ITG [10]. Our patient’s renal function and proteinuria improved after treating his CLL. Review of the literature suggests that some patients respond to prednisolone, cyclophosphamide, and other immunosuppressants alone or in combination. In cases where targeted clonal therapy is not available and these other agents are prescribed, it is not possible to determine with certainty if improved kidney function is due to direct immune suppression or a reduction of toxic CLL clones or both [11].

## Conclusions

Our patient had prior asymptomatic CLL and presented with the classic symptoms of MGRS and was found to have biopsy-proven ITG. Renal dysfunction resolved after treating the underlying CLL. This case highlights the need for screening for hematological malignancies in any patient who is diagnosed with ITG since the two have such a strong association. It is important to treat the underlying hematological disease for better long-term outcomes. Although a rare condition, hospitalists and primary care physicians should keep ITG in their differential diagnosis when patients present with hematuria, proteinuria, hypertension, and AKI. Monoclonal gammopathy of renal significance is a comparatively new classification that has opened the door for patients with monoclonal protein-associated renal disorders associated with hematologic conditions to be treated for their underlying disorder, even when oncologic therapeutic criteria have not been satisfied.
